# Upregulation of Derlin 3 (*DERL3*) protein expression is associated with Oral Cancer progression and is independent of promoter hypermethylation

**DOI:** 10.1186/s12885-026-15856-z

**Published:** 2026-03-13

**Authors:** Mayuri Inchanalkar, Rinal Chavda, Jitendra Gawde, Asawari Patil, Rajiv Kumar Kaushal, Manoj B. Mahimkar

**Affiliations:** 1 Mahimkar Lab, Cancer Research Institute, Advanced Centre for Treatment, Research and Education in Cancer (ACTREC), Tata Memorial Centre, Kharghar, Navi Mumbai, Maharashtra 410210 India; 2 Homi Bhabha National Institute, Training School Complex, Anushakti Nagar, Mumbai, Maharashtra 400094 India; 3 Biostatistician, Cancer Research Institute, Advanced Centre for Treatment, Research and Education in Cancer (ACTREC), Tata Memorial Centre (TMC), Kharghar, Navi Mumbai India; 4 Department of Pathology, Tata Memorial Hospital, Tata Memorial Centre, Mumbai, India

**Keywords:** Leukoplakia, OSCC, *DERL3*, Methylation, Gene expression, Copy number alteration, Protein expression, Disease progression

## Abstract

**Background:**

*DERL3* (Derlin 3) is a Derlin family protein involved in endoplasmic reticulum (ER) associated degradation of unfolded or misfolded glycoproteins. Our previous comprehensive genomic and transcriptomic profiling of leukoplakia and oral squamous cell carcinoma (OSCC) revealed amplification at the 22q11.23 locus, which harbours the *DERL3* gene. However, *DERL3* mRNA expression was found to be downregulated during disease progression, suggesting the role of epigenetic regulation.

**Methods:**

We evaluated promoter methylation using the Quantitative real-time methylation-specific PCR technique, copy number alterations using TaqMan assays, and protein expression through immunohistochemistry. The methylation levels between normals, leukoplakia and OSCC were compared using the Kruskal–Wallis test. Correlations between different techniques were computed using Spearman's rank correlation. The indirect and direct effects of *DERL3* gene amplification on methylation and protein expression were evaluated using mediation analyses. Recurrence-free survival and disease-specific survival were calculated using a log-rank test.

**Results:**

We observed a sequential, significant increase in promoter methylation of leukoplakia, early-stage, and advanced-stage OSCC (*p*-value < 0.001), compared to control tissues. The promoter methylation was inversely correlated with *DERL3* gene expression. Interestingly, the protein was overexpressed in leukoplakia (*p* = 0.023), early-stage (*p* = 0.012), and advanced-stage OSCC (*p* < 0.001) compared to normal tissue. High protein expression correlated with advanced-stage OSCC, pathological grade and habit profile, while methylation showed a significant association with age, pathological grade and advanced-stage OSCC. However, *DERL3* aberrations were not associated with patient survival.

**Conclusions:**

Our findings highlight that while promoter hypermethylation may account for reduced *DERL3* mRNA expression, the concomitant increase in DERL3 protein expression suggests that additional regulatory mechanisms contribute to OSCC progression.

**Supplementary Information:**

The online version contains supplementary material available at 10.1186/s12885-026-15856-z.

## Background

Oral squamous cell carcinoma (OSCC) is the most prevalent cancer in the world and is considered to derive from pre-existing oral lesions termed oral potentially malignant disorders (OPMDs). Leukoplakia is a predominant OPMD, having a malignant transformation rate ranging between 0.13% to 34% and is characterised by a white plaque/patch in the oral cavity [1]. The late-stage OSCCs are associated with high morbidity and mortality, with an overall 5-year survival rate around 40–50% due to locoregional recurrence and lymph node metastasis [2]. Along with age, tobacco abuse and alcohol consumption, human papillomavirus (HPV) infection also plays an important role in oral carcinogenesis. HPV infection is now considered a strong stratification factor associated with better prognosis and treatment de-escalation. Despite advances in treatment modalities, the prognosis of OSCC remains poor.

OSCC results from the complex interplay between genetic and epigenetic factors, and the exact molecular mechanism underlying oral carcinogenesis is unknown. It is a multifactorial process characterised by gain/hypomethylation of oncogenes and loss/hypermethylation of tumour suppressor genes that lead to recurrence, apoptosis resistance and metastasis. Our previous study identified copy number alterations (CNAs) and differential gene expression (GE) profiles associated with progression and clinical outcomes in HPV-negative OSCC [3]. We identified one interesting candidate gene, *DERL3* (Derlin-3) present on the 22q11.23 locus, which has been amplified; however, its expression is downregulated during disease progression. *DERL3*, a member of the Derlin family, plays a vital role in endoplasmic reticulum (ER) stress-induced ER-associated degradation (ERAD) for misfolded glycoproteins, which is critical for maintaining homeostasis [4]. The Derlin family members form an export channel in the membrane of the ER through which the misfolded protein substrates are transported for proteasomal degradation [5, 6]. The *DERL3* gene expression was validated by quantitative real-time polymerase chain reaction (qRT-PCR) in leukoplakia and OSCC and found to be negatively associated with disease progression [3]. The *DERL3* transcript downregulation was not a result of mutations because *DERL3* gene mutations have not been reported in head and neck cancers (Fig. S1) and may implicate the role of epigenetic regulations. Lopez-Serra et al. [7] demonstrated that it acts as a tumour suppressor gene in colon cancer, and *the DERL3* promoter was methylated in solid tumours, including head and neck cancers. However, a comprehensive analysis of *DERL3* promoter methylation, gene expression and protein expression altogether in OSCC has not been thoroughly investigated. In the present study, we aim to investigate and comprehensively understand the methylation status, CNAs, mRNA and protein expression in an independent validation set of clinically well-annotated leukoplakia and OSCC patients. Comprehending the mechanisms underlying OSCC progression may facilitate the discovery of novel therapeutic strategies.

## Materials and methods

### Sample collection

**Fig. 1:**
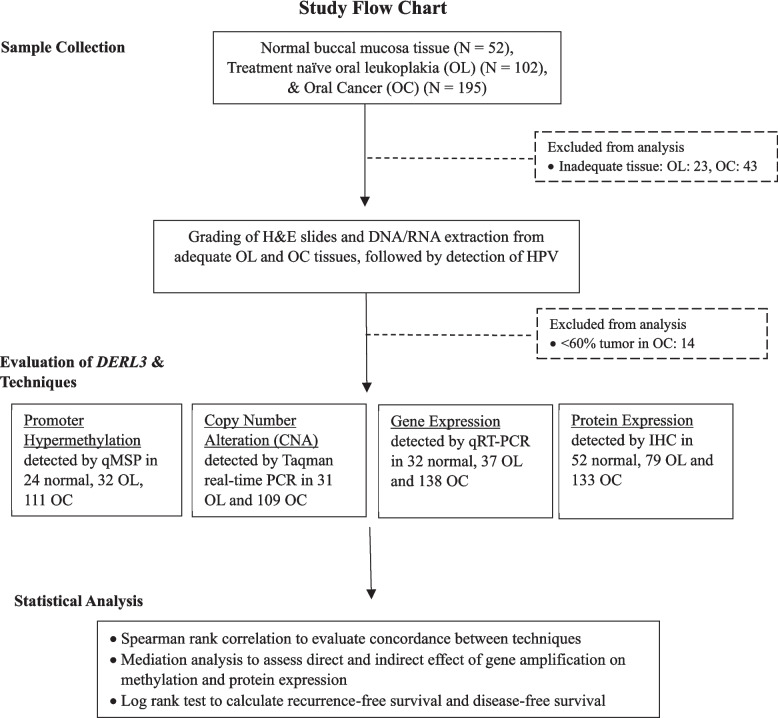
Overall workflow of study

Fresh frozen and formalin-fixed paraffin-embedded (FFPE) tissues of treatment-naive, surgically resected and pathologically diagnosed gingivobuccal OSCC (*N* = 195) and leukoplakia samples (*N* = 102) were recruited from Tata Memorial Hospital. Non-inflamed normal buccal mucosa tissues (*N* = 52) from clinically healthy individuals with no previous history of cancer were recruited from Nair Hospital Dental College. The inclusion criteria were (i) all stages of surgically resectable primary gingivobuccal cancers (with/without nodal metastasis) with confirmed histopathological grading and a tumor content of more than 60%, (ii) must not have received any treatment (Chemotherapy and/or Radiotherapy) before the recruitment and sample collection, (iii) gingivo buccal leukoplakia with confirmed hyperplasia and/or dysplasia histological grading. The study was approved by the Institutional Ethics Committee (IEC approval no 218 of 2016) of Tata Memorial Hospital and Nair Hospital Dental College, Mumbai, India. All procedures were carried out in accordance with the principles of the Declaration of Helsinki and the guidelines for good clinical practice. Written informed consent was obtained from all the study participants. Cryosectioning and Haematoxylin and Eosin (H&E) staining were performed, and the tumour content was assessed by two pathologists independently. The OSCC tissues having more than 60% tumour content and leukoplakia with hyperplastic and dysplastic histology were subjected to DNA and RNA extraction. The overall workflow of study is provided in Fig. 1.

### DNA extraction and quality control

DNA was extracted from frozen tissues using AllPrep® DNA/RNA/miRNA Universal kit (Cat no: 80224, Qiagen, Germany) according to the manufacturer’s instructions. Briefly, 25–30 mg of frozen tissue was crushed in a mortar and pestle using liquid nitrogen and buffer RLT Plus (Qiagen, Germany). The lysate was then homogenised using the RNase-free syringe, and nucleic acids were purified using the columns provided in the kit. The DNA and RNA were eluted in 100 µl and 50 µl Elution buffer (Qiagen, Germany), respectively. The nucleic acid was quantified through the NanoDrop-1000 spectrophotometer (Thermo Fisher Scientific). The integrity of DNA was checked using 2% Agarose gel electrophoresis, and amplifiability was determined by β-globin PCR.

### Detection of Human Papillomavirus (HPV)

The presence of HPV was detected by nested PCR [8]. This method involves two sets of primers, MY09/11 and GP5 + /GP6 + , which target the conserved region of the viral capsid protein and detect a wide range of high and low-risk HPV subtypes. The first round of PCR using MY09/11 produced a 450 bp product, which was used as a template for the second round of PCR with GP5 + /GP6 + primers, yielding a 150 bp product. HeLa cell line DNA was used as a positive control. Negative control was included in all the PCR reactions.

### Bisulfite treatment

Bisulfite conversion of the genomic DNA was performed using the EZ DNA Methylation-Direct kit (Zymo Research, Irvine, CA, USA) according to the manufacturer’s instructions. Briefly, 130 μL of CT conversion reagent was added to 400 ng of DNA, and subjected to bisulfite conversion in a Thermal cycler (Veriti, Applied Biosystems, California, USA). Bisulfite-converted DNA was purified using Zymospin columns provided in the kit and eluted in 25ul of M-Elution Buffer. For long-term storage, converted DNA was stored at -20^0^C. Universal methylated (Zymo Research, Irvine, CA, USA, Cat no: D5011-1) and unmethylated DNA (Qiagen, Germany, Cat no 59568) were used as positive and negative controls, respectively and followed the same bisulfite treatment conditions.

### Quantitative real-time methylation-specific PCR

The predesigned unmethylated and methylated primers specific for the *DERL3* promoter region [7] from Eurofins Genomics India Pvt Ltd were used for analysis. The quantitative real-time methylation-specific PCR** (**qMSP) was performed using the POWER SYBR Green PCR Kit (Qiagen, Germany). Briefly, 6.25 ng of bisulfite-treated DNA was used, and PCR reactions were performed in duplicates in a 384-well PCR plate. All batches included a universal methylated, unmethylated DNA plate control. PCR amplification of template DNA included initial denaturation at 95^0^C for 10 min; amplified for 40 cycles at 95^0^C for 10S and 57^0^C for 1 min, followed by melt curve analysis. The β-actin primers were used for normalisation and internal control. The percentage of promoter methylation was calculated as: methylated gene percentage 1/ [1 + 2.^(−∆Ct)^] × 100%, where ∆Ct is the difference between unmethylated and methylated Ct values (∆Ct = Ct_U_-Ct_M_) [9].

### Detection of *DERL3* Copy Number

The relative quantification of *DERL3* gene copy numbers was detected by TaqMan real-time PCR (Applied Biosystems: Hs00299710_cn) with female pooled DNA as the positive control. All the reactions were performed in quadruplets with 10 ng of DNA per reaction. The VIC and TAMRA dye-labelled *RNaseP* (located on chromosome 14q11.2) was used as an endogenous control, which has exactly two copies of the diploid genome. The PCR conditions were as follows: 95^0^C initial denaturation step for 10 min, 40 cycles at 95^0^ C for 15 s and 60^0^C for 1 min. The copy number was calculated using CopyCaller software v2.1 (Applied Biosystems). The copy number values ranging between 1.5 to 2.5 were considered as no change, more than 2.5 as gain/amplification and less than 1.5 as loss/deletion.

### Immunohistochemistry (IHC)

The *DERL3* protein expression was analysed through IHC using Vectastain Universal Elite ABC Kit (Vector Laboratories, USA). Five-micron FFPE sections were deparaffinized and rehydrated using xylene in a series of alcohols, followed by heat-based antigen retrieval in the EDTA buffer for 12 min. The non-specific binding was prevented by peroxidase and 1% serum blocking. Sections were incubated overnight at 4 °C with the primary antibody (Abcam, Cat no: ab78233). These sections were next treated with biotinylated secondary antibody solution and incubated with Vectastain Elite ABC reagent. The immunoreaction in tissue sections was visualised using 3, 3’-diaminobenzidine tetrahydrochloride hydrate (Sigma-Aldrich, MO). The counterstaining was performed with haematoxylin, and the slides were mounted. Sections treated with normal rabbit serum were used as negative controls. The cytoplasmic expression was graded by pathologists who were blinded to clinical outcomes for staining intensity and percentage of cells stained. The H-score (range, 0–300) was computed as follows: H-score was calculated as ΣPi (i), where Pi is the fraction of stained tumour cells (0–100%) at each intensity, and i is the staining intensity (on a scale of 0–3).

### Statistical analysis

The methylation levels between normals, leukoplakia and OSCC were compared using the Kruskal–Wallis test, followed by post hoc pairwise comparisons with adjusted p-values to account for multiple testing. Correlations/Interactions between different techniques were computed using Spearman's rank correlation. Mediation analysis was performed to assess the effect of *DERL3* gene amplification on methylation and protein expression. Both the average direct effect (ADE) and the average causal mediation effect (ACME), indirect effect, were estimated. The association of *DERL3* promoter hypermethylation, CNAs, gene expression, and protein expression with clinicopathological parameters was performed using the Chi-square test or Fisher’s Exact test. Recurrence-free survival and disease-specific survival were evaluated using a log-rank test. All the statistical analyses were performed with SPSS version 25 software (SPSS Inc., Chicago, IL, USA) and R 4.2.0 version. A p-value of < 0.05 was considered statistically significant.

## Results

### Clinicopathological characteristics

The demographic details and clinicopathological parameters of the study samples are represented in Table 1. The overlapping samples of normal buccal mucosa, leukoplakia and OSCC used in this study are depicted in Fig S2. Most of the leukoplakia cases were histopathologically hyperplastic (*n* = 70) and dysplastic (*n* = 9). The early-stage OSCC cases were 73 (37.4%), and 122 (62.6%) belonged to the advanced-stage OSCC, of which 40% of cases were positive for lymph node metastasis. All the samples were negative for HPV-DNA, which was consistent with previous results [3, 10].

**Table 1:** Association of *DERL3* aberrations with demographic and clinicopathological parameters

	**Promoter methylation**			**Copy number alterations**			**Gene expression**			**Protein expression**		
**Variables**	**Hypomethylated**	**Hypermethylated**	*p-***value**	**No change**	**Gain**	*p-***value**	**Low**	**High**	*p-***value**	**Low**	**High**	*p-***value**
Age (Years)												
< = 48	44	24	0.001^*^	47	19	0.215	39	44	0.319	65	45	0.665
> 48	26	46		57	14		47	39		55	43	
Gender												
Female	12	19	0.143	24	6	0.537	23	14	0.131	19	16	0.598
Male	60	52		82	28		65	70		104	72	
Clinical status												
Leukoplakia	22	10	0.038^*^	24	7	0.956	21	16	0.462	53	27	0.011^*^
Early stage OSCC	26	26		38	13		25	31		34	18	
Advanced stage OSCC	24	35		44	14		42	37		36	44	
Pathological grade												
Hyperplasia	19	8	0.039^*^	21	6	0.571	18	14	0.569	44	26	0.035^*^
Mild dysplasia	1	2		3	0		1	2		4	0	
Moderate dysplasia	2	0		0	1		2	0		4	0	
Severe Dysplasia	NA	NA		NA	NA		NA	NA		1	0	
Well	3	9		10	2		8	4		8	7	
Moderate	34	33		50	16		41	46		38	43	
Poor	13	19		22	9		18	18		24	11	
Tumor stage												
T1/T2	37	42	0.551	55	27	0.154	40	49	0.130	49	31	0.019^*^
T3/T4	13	19		22	5		27	19		21	31	
Lymph node involvement												
N0	33	39	0.821	56	15	0.228	40	39	0.782	46	37	0.474
N +	17	22		26	12		27	29		24	25	
Habit profile												
Never	2	1	0.830	2	1	0.855	1	2	0.664	0	6	0.003^*^
Exclusive chewer	36	39		57	17		46	46		50	39	
Exclusive smoker	4	2		4	1		5	2		12	2	
Exclusive drinker	1	0		1	0		0	1		1	0	
Mixed Habit	18	18		25	11		22	19		48	27	
LVE												
No	40	57	0.418	71	24	1.000	52	60	0.222	68	61	1.000
Yes	1	0		1	0		2	0		2	1	
PNI												
No	36	53	0.485	64	23	0.443	47	56	0.256	62	51	0.302
Yes	5	4		8	1		7	4		8	11	
ECS												
No	33	44	0.695	58	18	0.562	46	44	0.121	58	47	0.642
Yes	8	13		14	6		8	16		12	12	

All the samples belong to gingivobuccal complex (GBC) of oral cavity, NA: Not applicable because of missing values, * Significance at *p* < 0.05

### *DERL3* promoter methylation status in leukoplakia and OSCC samples

**Fig. 2:**
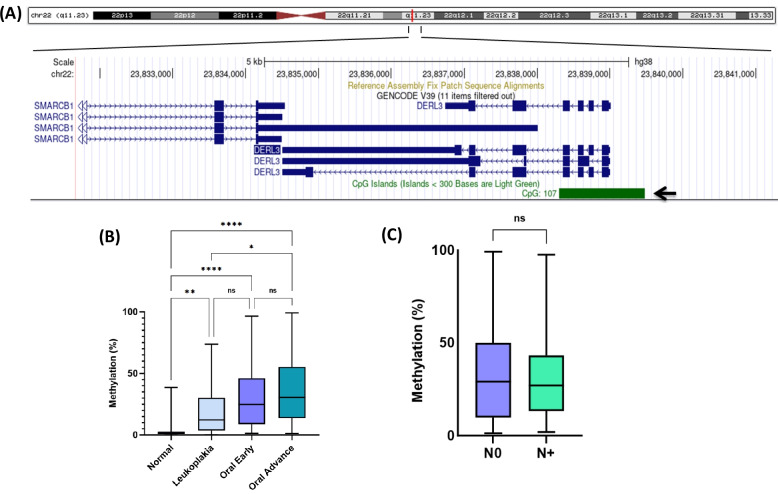
*DERL3* promoter methylation analysis (**A**) illustrates the presence of a CpG island (indicated by a black arrow) in the DERL3 promoter region retrieved from the UCSC genome browser. Boxplot showing Methylation (%) difference in (**B**) normal, leukoplakia, early and advanced stage OSCC and (**C**) node negative Vs node positive

Figure 2A shows the presence of a CpG island in the *DERL3* promoter region spanning over 4 exons. We analysed *DERL3* promoter methylation in 24 normal buccal mucosa (control), 32 leukoplakia and 111 OSCC samples using the qMSP technique. In comparison to control tissues (1%), there was a sequential increase in the methylation levels of leukoplakia (Median 12.5%, *p* = 0.003), early (25%, *p* < 0.001) and advanced stage OSCC (31%, *p* < 0.001) (Fig. 2B). Similarly, in comparison to leukoplakia (12.5%), advanced stage (31%, *p* = 0.011) OSCCs showed higher methylation status. However, there was no significant methylation difference between leukoplakia vs early stage OSCC (*p* = 0.272), early vs advanced stage OSCC (*p* = 0.156) and node-positive vs node-negative patients (*p* = 0.951) (Fig. 2C). Further, to confirm our results, evaluation of The Cancer Genoma Atlas (TCGA) dataset from the University of ALabama at Birmingham CANcer data analysis portal platform [11] was done, which revealed hypermethylation of the *DERL3* promoter in primary head and neck squamous cell carcinoma (HNSCC) tumours (Fig. S3).

### Evaluation of CNAs in the *DERL3* gene located at the 22q11.23 locus

**Fig. 3:**
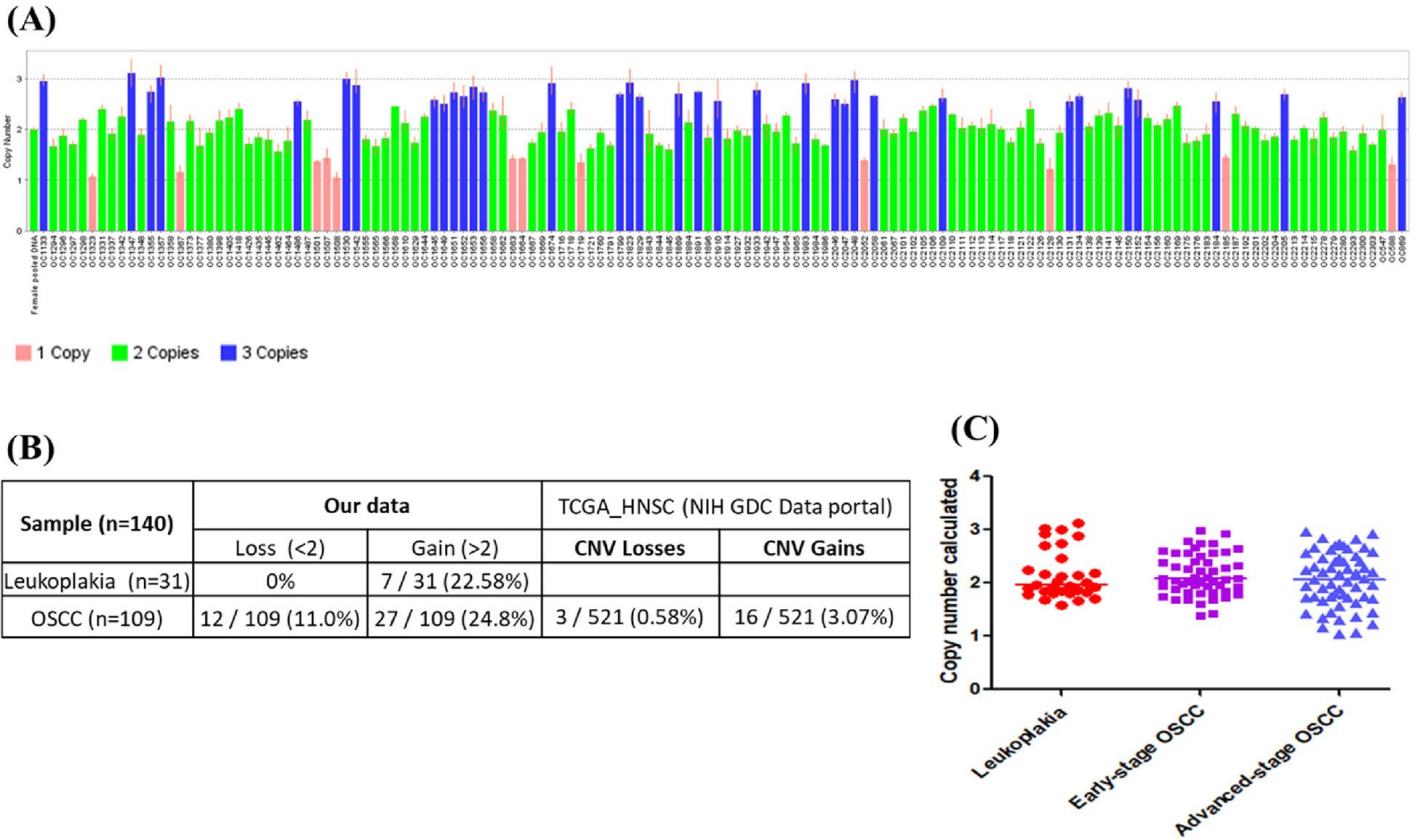
*DERL3* copy number alterations (**A**) *DERL3* copy number calculated by CopyCaller software (**B**) Summary of CNAs in leukoplakia and OSCC (**C**) Frequency of CNV alterations in leukoplakia, early and advanced stage OSCC

We analysed CNAs in 31 leukoplakia and 109 OSCC samples using TaqMan CNA assays. The CNAs were observed in both leukoplakia (22.58%) and OSCC (35.8%) (Fig. 3A). Seven out of thirty-one (22.6%) leukoplakia samples showed 3 gene copies, and no deletions/losses were observed. CNAs for OSCC were observed in 39 (35.8%) individuals, with 27 (24.8%) samples having 3 gene copies and 12 (11%) samples having a single gene copy number (Fig. 3B and 3C). We did not observe complete gene loss in any leukoplakia or OSCC samples. The HNSCC TCGA data from the Genomic Data Commons data portal reports 3.07% copy number gains and 0.58% copy number losses.

### *DERL3* protein expression in leukoplakia and OSCC

**Fig. 4:**
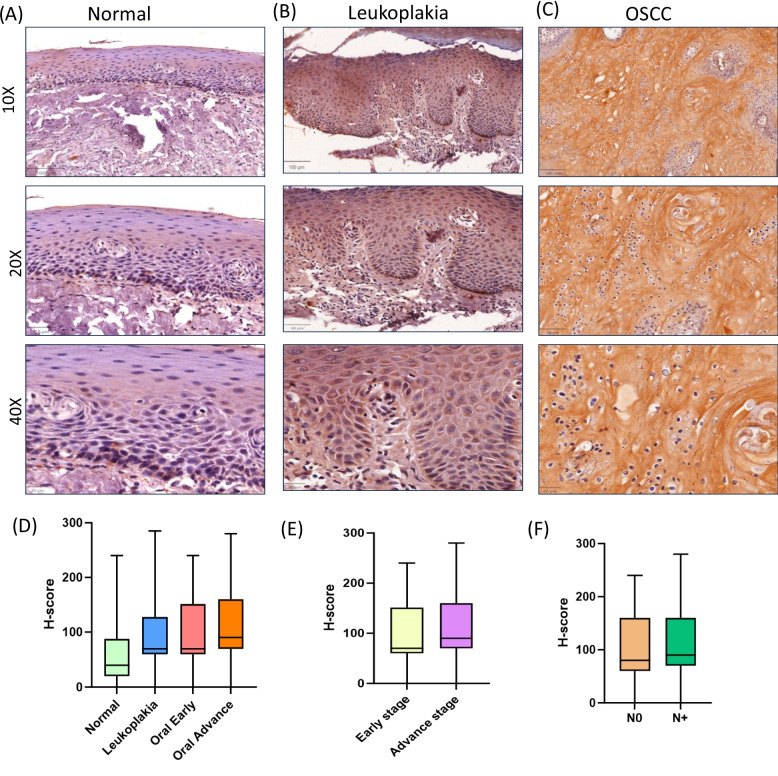
*DERL3* protein expression analysis. **B** Leukoplakia and (**C**) OSCC tissues showed strong cytoplasmic staining as compared to (**A**) normal buccal mucosa. Boxplot (**D**) showing a gradual increase in protein expression as the lesion progresses. **E** A significant increase in protein levels was observed in advanced-stage OSCC compared to early-stage OSCC. However, no difference was observed in (**F**) node-positive vs node-negative samples

Using IHC, the protein expression was analysed in 52 normal, 79 leukoplakia and 133 OSCC tissue samples. Strong cytoplasmic expression was observed in leukoplakia (Fig. 4B) and OSCC (Fig. 4C) tissues compared to weak staining in normal buccal mucosa (Fig. 4A). A statistically significant higher protein expression was observed in leukoplakia (*p* = 0.023), early (*p* = 0.012), and advanced-stage OSCC (*p* < 0.001) compared to normal. Similarly, advanced-stage OSCC had higher protein levels compared to leukoplakia (*p* = 0.007) and early-stage OSCC (*p* = 0.04).

### Correlation/Interaction between promoter methylation, CNAs, gene expression and protein expression

**Fig. 5:**
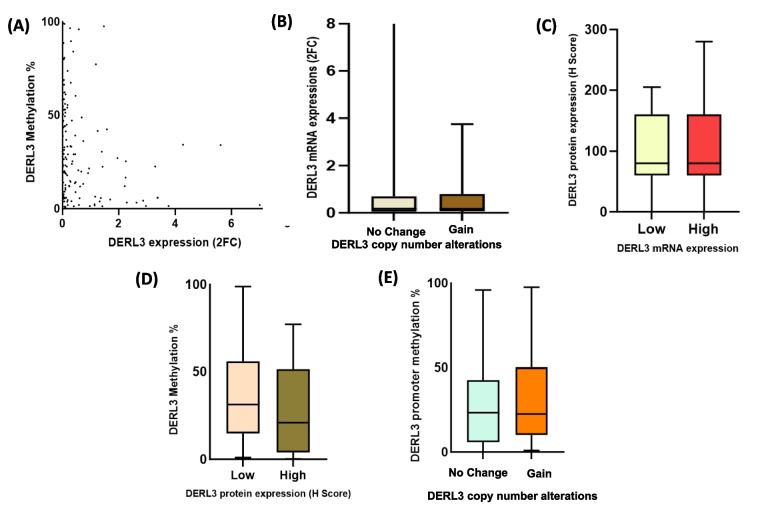
Correlation analyses. Scatterplot (**A**) depicts a negative correlation between *DERL3* promoter methylation and gene expression. Boxplots show no correlation between *DERL3* gene expression with (**B**) CNAs and (**C**) protein expression. Similarly, *DERL3* promoter methylation did not have any effects on (**D**) protein expression and (**E**) copy number alterations

The gene expression of *DERL3* for the same set of samples was evaluated previously [3], which was correlated with the methylation status obtained in the current study to determine the biological significance. Table 2 represents the pairwise correlations between methylation, gene expression, CNA and protein expression using maximum common samples available for each assay combination; across normal, oral leukoplakia and OSCC. As presented in Fig. 5A, *DERL3* downregulation in OSCC is significantly associated with promoter hypermethylation, demonstrating a weak inverse correlation (r = − 0.295, *p* = 0.002). Next, we examined whether gene expression is correlated with CNAs and protein expression in OSCC. However, there was no significant correlation between gene expression with CNAs (r = 0.105, *p* = 0.282) and protein expression (r = -0.007, *p* = 0.955) (Fig. 5B and 5C). Similarly, there was no correlation between promoter methylation with CNAs (r = − 0.110, *p* = 0.257) and protein expression (r = − 0.193, *p* = 0.435) (Fig. 5D and 5E). The CNAs also had a weak correlation with protein expression (r = − 0.193, *p* = 0.142). In OL, no significant correlations were observed between methylation, gene expression, CNA, or protein expression (Table 2).

**Table 2:** Correlation between methylation, gene expression, copy number alterations, and protein expression across normal mucosa, oral leukoplakia, and OSCC

Parameters	Diagnostic Group	N	Correlation (r)	*p*-value
Methylation & Gene Expression	Normal	19	–0.109	0.658
	Leukoplakia	32	–0.149	0.416
	OSCC	109	–0.295**	0.002*
Methylation & CNA	Leukoplakia	31	–0.069	0.712
	OSCC	109	–0.110	0.257
Methylation & Protein Expression	Leukoplakia	14	–0.310	0.281
	OSCC	59	–0.104	0.435
CNA & Gene Expression	Leukoplakia	31	0.164	0.378
	OSCC	108	0.105	0.282
CNA & Protein Expression	Leukoplakia	13	0.299	0.320
	OSCC	59	–0.193	0.142
Gene Expression & Protein Expression	Leukoplakia	15	0.165	0.556
	OSCC	74	–0.007	0.955

^**^ = Significant at alpha = 0.01, * Significance at *p* < 0.05

The CNAs' (gene amplification) effect on methylation and protein expression was evaluated using mediation analyses. The CNA's effect on mRNA indirectly through promoter methylation (*n* = 139; overall common samples with methylation, CNA and, mRNA data) showed a non-significant association (ACME = 0.017, 95% CI –0.070 to 0.127; *p* = 0.650). Further, there was no direct effect (ADE = 0.029, 95% CI –0.424 to 0.375; *p* = 0.768) of CNA on the mRNA expression. Next, we assessed whether CNAs affect protein expression through mRNA levels (CNA → mRNA → protein expression) in *n* = 72 cases; overall common samples with CNA, mRNA and protein expression data. Neither the indirect effect (ACME = –0.012, 95% CI –2.40 to 5.37; *p* = 0.991) nor the direct effect (ADE = –7.25, 95% CI –36.6 to 24.8; *p* = 0.574) was significant, suggesting that *DERL3* copy-number changes do not exert measurable effects on protein expression through mRNA-mediated mechanisms in our cohort.

Overall, these findings demonstrate that *DERL3* downregulation in our cohort is primarily associated with promoter hypermethylation. In contrast, CNAs did not correlate with gene expression, promoter methylation or protein expression. Mediation analyses further confirmed that CNA exert neither direct nor indirect effect on mRNA or protein levels. Collectively, our results indicate that promoter hypermethylation rather than CNA, contributes to the reduced *DERL3* gene expression, while the increased protein expression observed in our cohort reflects the influence of additional complex regulatory mechanisms beyond trasncriptional control.

### Relationship between *DERL3* aberrations with clinicopathological parameters and Survival analysis

The relation between *DERL3* aberrations and clinicopathological characteristics is summarized in Table 1. A significant correlation was observed between *DERL3* methylation and age (*p* = 0.001), pathological grade (*p* = 0.039) and advanced-stage OSCC (*p* = 0.038), high *DERL3* protein expression was significantly correlated with advanced-stage OSCC (*p* = 0.032), pathological grade (*p* = 0.035) and habit profile (*p* = 0.003). No significant associations were detected between *DERL3* CNAs and mRNA expression with other clinicopathological parameters.

In the survival analysis, we did not find any correlation between *DERL3* aberrations with recurrence-free survival and disease-specific survival (Table S1), suggesting that *DERL3* dysregulation may contribute primarily to tumour initiation and progression rather than directly impacting long-term patient prognosis.

## Discussion

In this study, we comprehensively investigated the regulatory mechanisms influencing *DERL3* expression by evaluating promoter methylation, CNAs and protein expression patterns in leukoplakia and OSCC tissue samples. Promoter DNA methylation and CNAs are recognised as key regulatory mechanisms that predominantly affect gene transcription and, consequently, mRNA and protein expression patterns [12]. We observed a sequential significant increase in methylation as the disease progressed from leukoplakia to OSCC, which showed a significant inverse correlation with mRNA expression. As reported previously, CNAs did not have any effects on gene expression; however, there was an increase in protein expression, suggesting alternative mechanisms regulating protein expression [3]. The clinicopathological parameters, including age and habit profile, were significantly associated with *DERL3* hypermethylation and protein expression respectively whereas advanced stage OSCC and pathological grade was associated with both. However, no significant correlation was observed between *DERL3* aberrations and patient survival. Thus, while promoter hypermethylation contributes to reduced gene expression, it does not influence the observed increase in *DERL3* protein expression, implying regulation through alternative pathways.

Chromosomal aberrations are one of the key hallmarks of cancer, and multiple studies have focused on elucidating the abnormalities associated with disease progression. The regional copy number gains in 22q11.23 have been reported in a variety of tumors [13, 14] and found to be associated with poor prognosis and affecting the imatinib response in chronic myeloid leukaemia [15]. Interestingly, the loss in the 22q11.23 region was also observed in many cancers, including OSCC [16] and oral tongue carcinoma [17]. In gastric carcinoma, loss in this region was associated with lymph node metastasis [18, 19]. Previously, Bhosale et al. [3] profiled CNAs and gene expression in leukoplakia and OSCC, and found an opposite trend, i.e. significant expression change (downregulation) in the *DERL3* gene in an amplified 22q11.23 region, suggesting that *DERL3* is an intrinsic gene with its expression regulated independent of CNAs.

Unfolded protein response (UPR) signalling contributes to cancer progression and has been associated with multiple hallmarks of malignancy, including inflammation, angiogenesis, genomic instability, invasion, proliferation, survival, and apoptosis resistance [20]. *DERL3* (located on 22q11.23) is a component of ERAD and is upregulated in the UPR pathway. In several tumors, *DERL3* silencing has been linked to aggressive biological behaviour, supporting its role as tumor suppressor. *DERL3* is frequently downregulated through epigenetic inactivation in solid tumours and human malignant melanomas [21, 22], contributing to the metabolic reprogramming and Warburg effect [7]. Consistently, in gastric cancer, promoter hypermethylation of *DERL3* has been associated with reduced gene expression and tumor progression [22]. In colorectal cancer, Yu et al. demonstrated that *DERL3* is significantly downregulated, and its overexpression supresses cell proliferation, migration and invasion, further supporting tumor suppressive activity [23]. Similarly, repression of *DERL3* via DNA methylation by Epstein-Barr virus latent membrane protein in nasopharyngeal carcinoma leads to increased proliferation and invasion, suggesting that loss of *DERL3* contributes to tumor progression [24]. Conversely, *DERL3* has also been implicated in oncogenic behaviour in tumors that are highly dependent on ER stress adaptation. Under such conditions, *DERL3* upregulation supports enhanced ER proteostasis and cell survival. Shibata et al. [25] reported that *DERL3* overexpression promotes breast cancer progression and is associated with malignant phenotype and poor prognosis. Further, in lung adenocarcinoma, *DERL3* is significantly upregulated and associated with poor therapeutic response, suggesting a pro-tumorigenic role in stress-adapted microenvironments [26]. These findings highlight that *DERL3* exhibits context-dependent dual functionality. In line with this, our study suggests that *DERL3* protein upregulation in OSCC may reflect a stress-adaptive, pro-survival mechanism, despite mRNA downregulation.

DNA methylation is one of the major epigenetic mechanisms to regulate gene expression. Hypermethylation of tumour suppressor genes and hypomethylation of oncogenes are among the earliest epigenetic events in carcinogenesis, driving tumour initiation, disease progression and metastasis. Abe et al. reported aberrant promoter methylation of multiple genes in high-risk oral leukoplakia [27]. Further, we identified an increase in aberrant methylation as the lesion progresses from OPMD to OSCC [28]. The gene-specific methylation at CpG islands within or near the promoter region tightly regulates gene expression [29]. The *DERL3* promoter region encompasses a CpG island containing 107 CpG sites spanning over 4 exons, which may indicate regulation by DNA methylation. We observed that *DERL3* promoter methylation increased sequentially during the progression from leukoplakia to OSCC, suggesting its potential role in malignant transformation. In addition, *DERL3* methylation level is negatively correlated with its mRNA expression level, suggesting that *DERL3* mRNA expression was regulated by DNA methylation. The TCGA-HNSCC samples also revealed hypermethylation of the *DERL3* promoter, confirming our results. Consistent with our findings, Li et al. reported low mRNA expression of *DERL3* in gastric tumours, which was found to be associated with the epigenetic regulation mechanism in the development of gastric cancer. They further validated this observation through functional assays—cell proliferation, cell scratch, and cell invasion and confirmed that *DERL3* acts as a tumour suppressor gene inhibiting the malignant evolution of gastric cancer [22]. Similarly, Serra et al. reported that *DERL3* CpG island hypermethylation leads to gene inactivation in colorectal tumorigenesis and highlights the tumour suppressor features of the *DERL3* gene [30]. Next, we also validated CNAs and protein expression, but did not observe any direct or indirect correlation with gene expression and promoter methylation.

Interestingly, we observed a significant increase in *DERL3* protein expression, although the mRNA transcript was downregulated, suggesting alternative mechanisms regulating protein expression. Such mRNA-protein disconcordance is well documented in cancer biology. Zerdes et al., reported a weak correlation between PDL1 mRNA and protein levels in early breast cancer [31]. Similarly, in prostate cancer progression, proteomic profiles do not match genomic or transcriptomic alterations, indicating that proteomic regulation can operate independently and in some cases reflect disease state more accurately than mRNA levels alone [32]. Furthermore, Cheng et al. examined proteomic and transcriptomic dynamics in mammalian cells exposed to dithiotheritol ER stress over a 30-h period. Their findings showed that mRNA levels exhibited transient fluctuations and eventually returned to baseline values. In contrast, protein levels underwent a single sustained shift and remained stable thereafter, highlighting that protein abundance during misfolding stress is regulated by mechanisms beyond transcription alone, although the exact mechanisms were not delineated in their study [33].

The possible underlying reasons might be multiple layers of post-transcriptional regulation, such as splicing, polyadenylation, nuclear export, mRNA storage, and stability, all of which can modulate protein abundance independent of transcript levels [34]. Moreover, activation of UPR during ER stress is known to generate disconcordance between mRNA and protein levels in tumor cells. UPR signalling through Protein Kinase RNA-like endoplasmic reticulum like kinase (PERK), Inositol-requiring enzyme 1 alpha, and Activating transcription factor 6 reprograms translation and enhances ER proteostasis [35]. PERK-mediated eukaryotic initiation factor 2 alpha phosphorylation suppresses global translation but selectively enhances synthesis of stress adaptive proteins [36], enabling increased protein abundance even when transcription is reduced. ER stress also influences proteosomal turnover and ERAD pathways, potentially reducing degradation and increasing protein stability [36]. Additionally, hypoxia and nutrient stress within the tumour microenvironment can reshape translational programs, driving tumorigenesis and malignancy [37]. Further, the post-translational modifications database [38] reports potential phosphorylation sites (S89 and S136); however, functional validation is required. Together, these parameters support the observed increased protein expression despite downregulation of mRNA in our study. Although no significant association was observed between *DERL3* aberrations and other clinicopathological parameters and survival of the patient, high protein expression correlated with advanced stage, pathological grade and habit profiles. Collectively, these findings indicate that *DERL3* promoter hypermethylation represents an early event in oral carcinogenesis.

We acknowledge the limitations of the study, including a small sample size, single-centre cohort, and lack of functional validation (in vitro and in vivo studies) to mechanistically confirm the biological role of *DERL3* in OSCC. Despite this, to the best of our knowledge, this is the first study to comprehensively assess *DERL3* promoter methylation, CNAs, gene expression, and protein expression in an independent cohort of leukoplakia and OSCC patient samples. Multiple mechanisms, including epigenetic, transcriptional, and post-transcriptional, influence the expression of *DERL3*. While promoter methylation may act as an early silencing event, subsequent protein overexpression in association with advanced stage and habit profiles highlight a complex regulatory environment. These findings warrant the need for more thorough research to identify the molecular underpinnings of *DERL3* regulation and its potential as a biomarker in oral carcinogenesis.

## Conclusion

The current study demonstrates the role of CpG-island hypermethylation in deregulating the *DERL3* gene expression. Notably, *DERL3* protein expression is independent of hypermethylation, suggesting involvement of complex regulation of *DERL3* in oral carcinogenesis.

## Supplementary Information

Supplementary Material 1.

Supplementary Material 2.

## Data Availability

The data underlying this article is available in the article itself and in its online supplementary material.

## Abbreviations

OSCC: Oral Squamous Cell Carcinoma
OPMD: Oral Potentially Malignant Disorder
HPV: Human Papillomavirus
CNA: Copy Number Alterations
GE: Gene Expressions
qMSP: Quantitative real-time methylation-specific PCR
ER: Endoplasmic Reticulum
ERAD: ER Associated Degradation
UPR: Unfolded Protein Response
PERK: Protein Kinase RNA-like endoplasmic reticulum like kinase
