# Supplementary material for: Upregulation of Derlin 3 (DERL3) protein expression is associated with Oral Cancer progression and is independent of promoter hypermethylation
Source: BMC Cancer. 2026 Mar 13;26:513. doi: 10.1186/s12885-026-15856-z (PMC13104510; doi:10.1186/s12885-026-15856-z)
Supplement: Supplementary file 1 — Supplementary Material 1. [file 12885_2026_15856_MOESM1_ESM.pptx]

## Slide 1
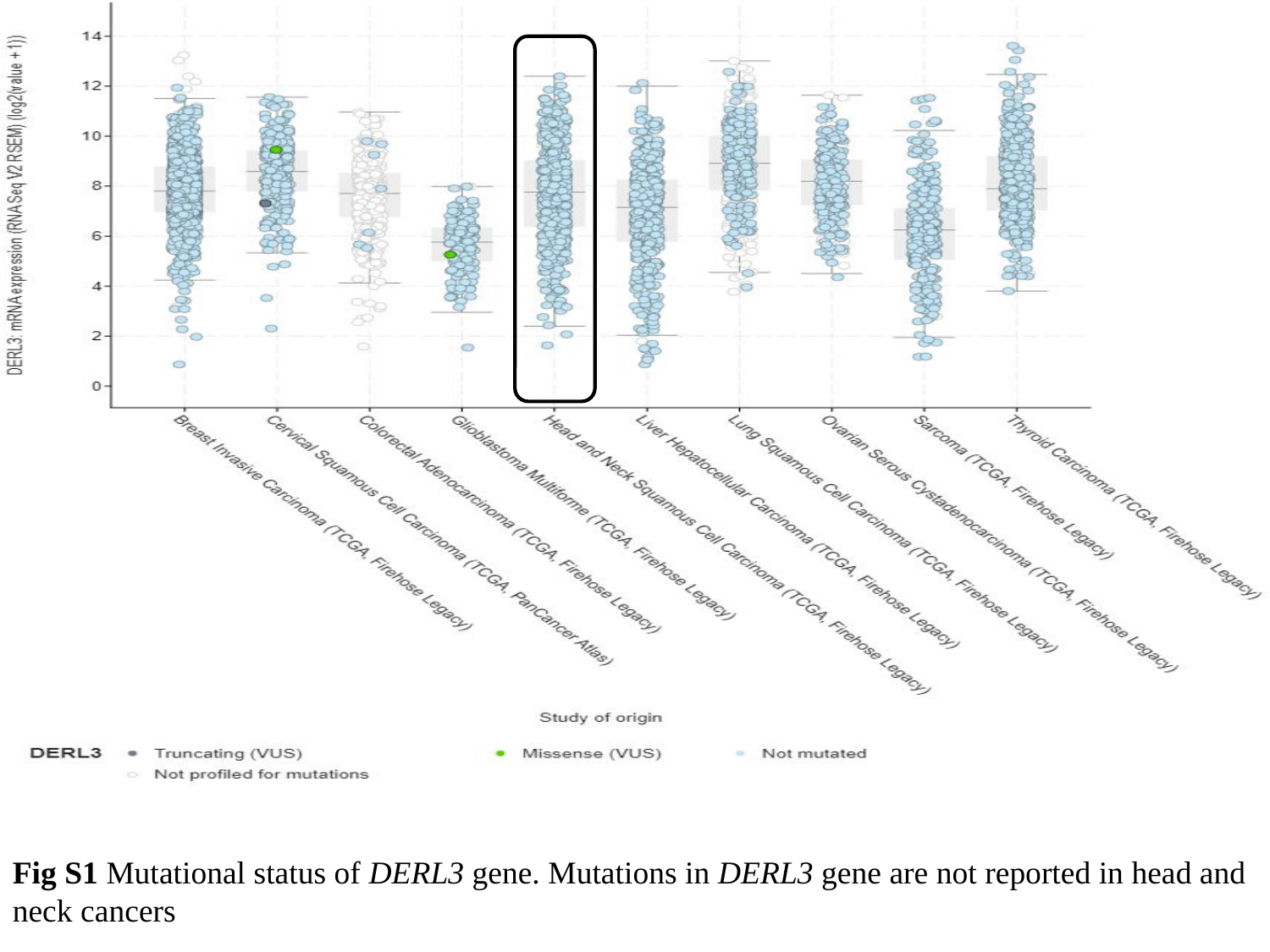

Fig S1 Mutational status of DERL3 gene. Mutations in DERL3 gene are not reported in head and neck cancers

## Slide 2
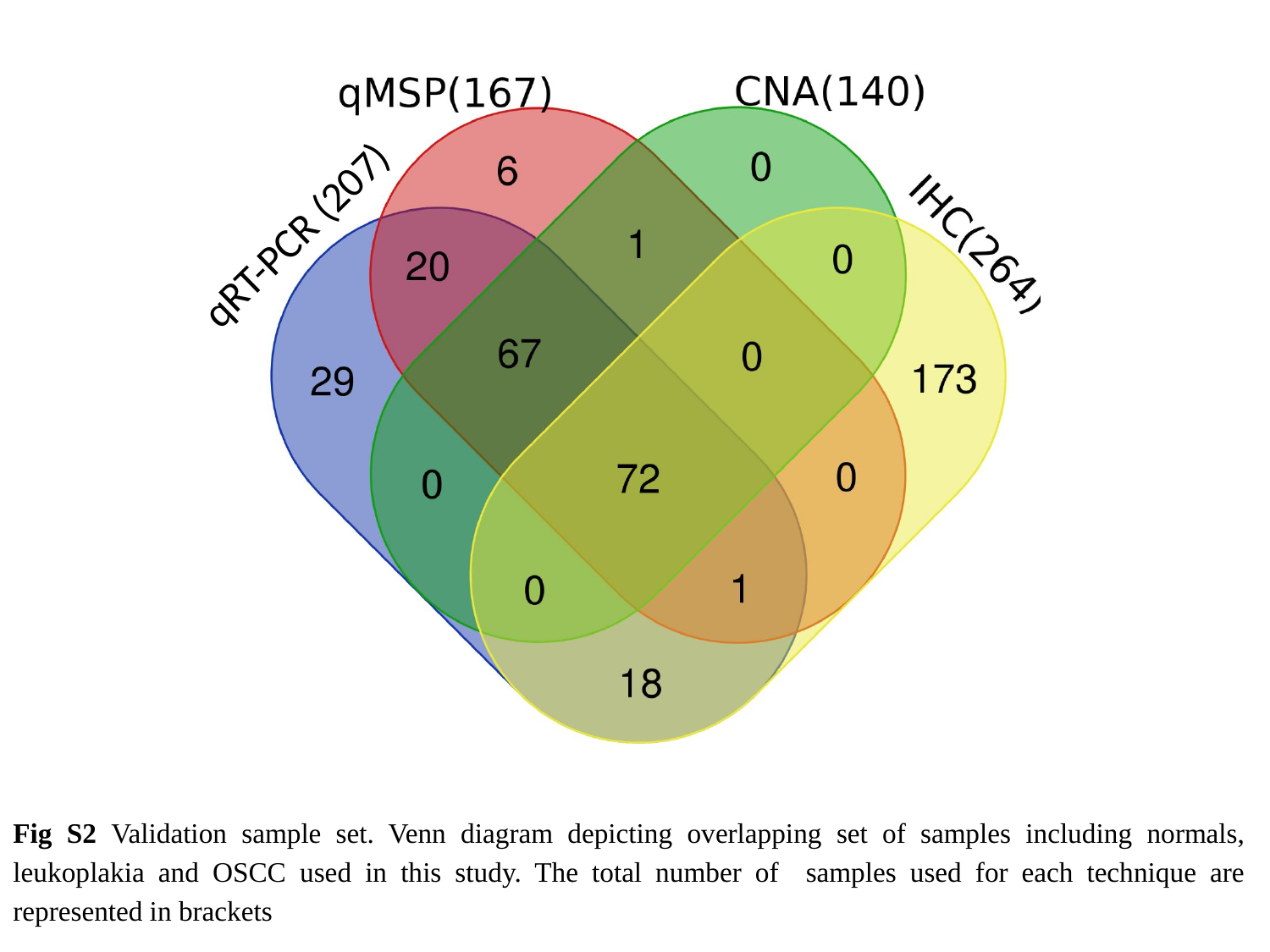

qRT-PCR (207)
Fig S2 Validation sample set. Venn diagram depicting overlapping set of samples including normals, leukoplakia and OSCC used in this study. The total number of samples used for each technique are represented in brackets

## Slide 3
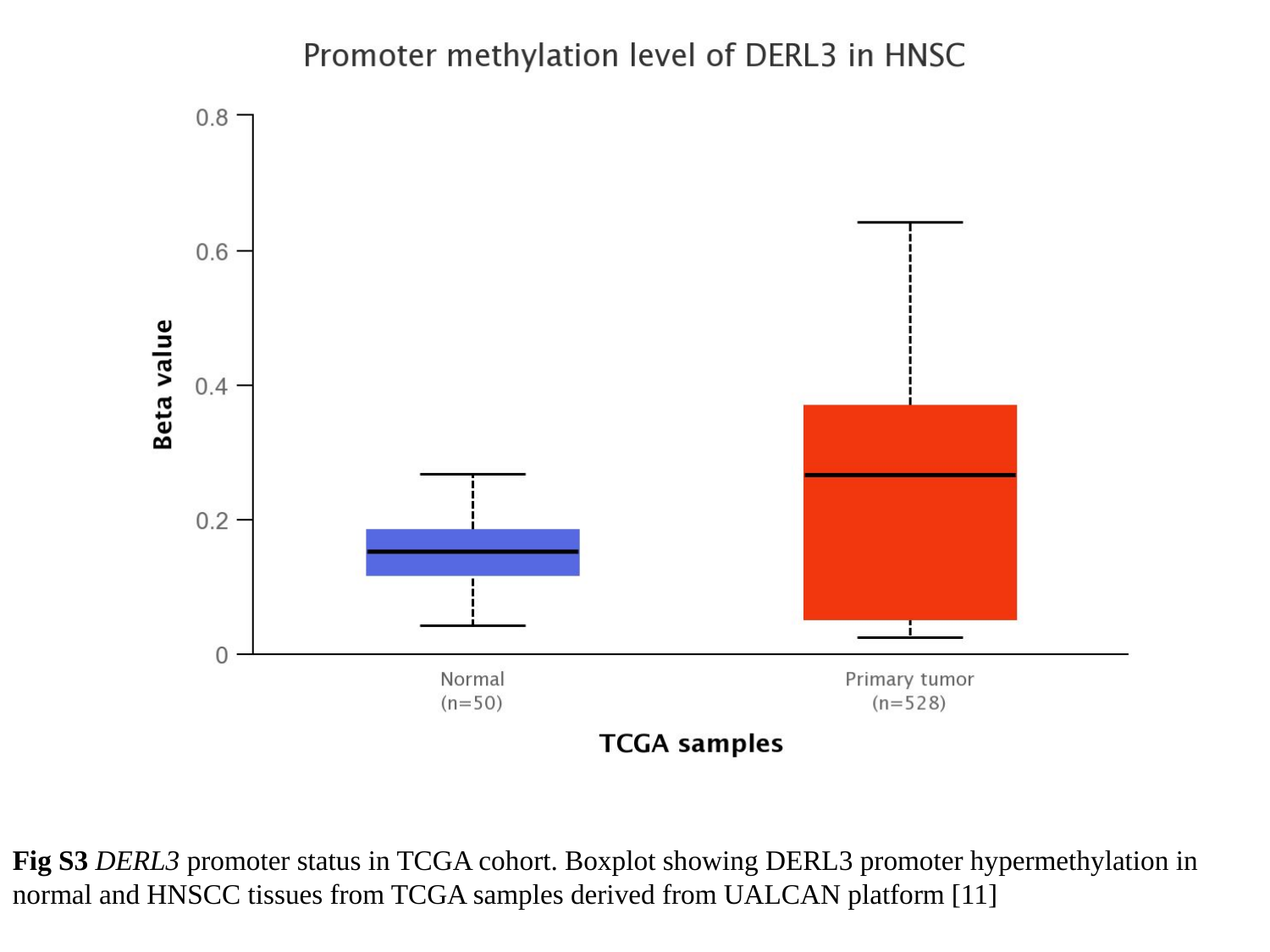

Fig S3 DERL3 promoter status in TCGA cohort. Boxplot showing DERL3 promoter hypermethylation in normal and HNSCC tissues from TCGA samples derived from UALCAN platform [11]
