# Supplementary material for: Upregulation of Derlin 3 (DERL3) protein expression is associated with Oral Cancer progression and is independent of promoter hypermethylation
Source: BMC Cancer. 2026 Mar 13;26:513. doi: 10.1186/s12885-026-15856-z (PMC13104510; doi:10.1186/s12885-026-15856-z)
Supplement: Supplementary file 2 — Supplementary Material 2. [file 12885_2026_15856_MOESM2_ESM.pdf]

Table S1: Kaplan-Meier analysis of *DERL3* aberrations and clinical outcome

Variables		Recurrence free survival (RFS)		Disease specific survival (DSS)	
		**Total N (Events)**	***P-value***	**Total N (Events)**	***P-value***
Promoter methylation	Hypomethylated	50 (23)	0.932	49 (9)	0.234
	Hypermethylated	48 (23)		46 (13)	
Copy number alterations	No change	75 (32)	0.080	73 (15)	0.166
	Gain	21 (14)		20 (7)	
Gene expression	Low	63 (30)	0.819	59 (14)	0.653
	High	65 (39)		64 (17)	
Protein expression	Low	51 (23)	0.739	50 (8)	0.880
	High	68 (31)		65 (11)	
